# (*S*)-2-[1-(4-Bromo­phen­yl)-1-hy­droxy­ethyl]-5,5-dimethyl-1,3,2-dioxaphos­phinane 2-oxide

**DOI:** 10.1107/S1600536811020587

**Published:** 2011-06-18

**Authors:** Chubei Wang, Hao Peng, Hongwu He

**Affiliations:** a Key Laboratory of Pesticide and Chemical Biology, College of Chemistry, Central China Normal University, Wuhan 430079, People’s Republic of China.

## Abstract

In the crystal structure of the title mol­ecule, C_13_H_18_BrO_4_P, the phospho­nate ring adopts a chair conformation. Mol­ecules are linked by an O—H⋯O hydrogen bond [O⋯O = 2.780 (3) Å] to form chains parallel to the *c* axis. Two C—H⋯O inter­actions help to stabilize the crystal structure.

## Related literature

For the synthesis and biological activity of hy­droxy­phospho­nate derivatives, see: Peng *et al.* (2007 ▶); Liu *et al.* (2006 ▶). For the synthesis of hy­droxy­phospho­nates, see: Zhou *et al.* (2008 ▶). For standard bond lengths, see: (Allen *et al.*, 1987 ▶).
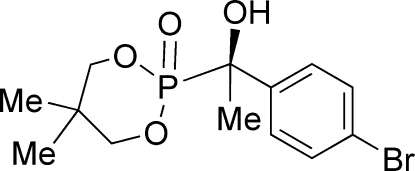


## Experimental

### 

#### Crystal data

C_13_H_18_BrO_4_P
*M* *_r_* = 349.15
Orthorhombic, 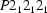

*a* = 11.0662 (17) Å
*b* = 11.3149 (18) Å
*c* = 11.9609 (19) Å
*V* = 1497.7 (4) Å^3^
*Z* = 4
Mo *K*α radiation
μ = 2.86 mm^−1^
*T* = 298 K
0.20 × 0.12 × 0.10 mm

#### Data collection

Bruker SMART APEX CCD area-detector diffractometer
10072 measured reflections
3627 independent reflections
2691 reflections with *I* > 2σ(*I*)
*R*_int_ = 0.114

#### Refinement

*R*[*F* ^2^ > 2σ(*F* ^2^)] = 0.045
*wR*(*F* ^2^) = 0.105
*S* = 0.93
3627 reflections
176 parameters
H-atom parameters constrained
Δρ_max_ = 0.46 e Å^−3^
Δρ_min_ = −0.45 e Å^−3^
Absolute structure: Flack (1983 ▶), with 1517 Friedel pairs
Flack parameter: −0.011 (10)

### 

Data collection: *SMART* (Bruker, 2001 ▶); cell refinement: *SAINT-Plus* (Bruker, 2001 ▶); data reduction: *SAINT-Plus*; program(s) used to solve structure: *SHELXS97* (Sheldrick, 2008 ▶); program(s) used to refine structure: *SHELXL97* (Sheldrick, 2008 ▶); molecular graphics: *PLATON* (Spek, 2009 ▶); software used to prepare material for publication: *PLATON*  and *SHELXL97*.

## Supplementary Material

Crystal structure: contains datablock(s) global, I. DOI: 10.1107/S1600536811020587/qk2003sup1.cif

Structure factors: contains datablock(s) I. DOI: 10.1107/S1600536811020587/qk2003Isup2.hkl

Supplementary material file. DOI: 10.1107/S1600536811020587/qk2003Isup3.cml

Additional supplementary materials:  crystallographic information; 3D view; checkCIF report

## Figures and Tables

**Table 1:** Hydrogen-bond geometry (Å, °)

*D*—H⋯*A*	*D*—H	H⋯*A*	*D*⋯*A*	*D*—H⋯*A*
O1—H1⋯O2^i^	0.82	2.01	2.780 (3)	156
C9—H9*B*⋯O1	0.97	2.58	3.163 (4)	119
C11—H11*A*⋯O2^i^	0.97	2.57	3.517 (4)	165

Symmetry code: (i) 
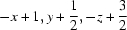
.

**Fig. 1.**
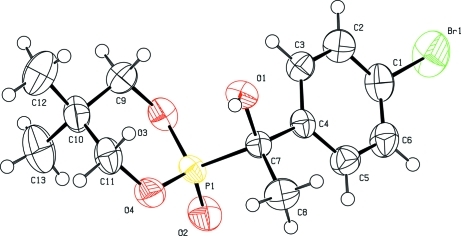
Molecular structure of (I), showing the labeling scheme and 50% probability thermal ellipsoids.

**Fig. 2.**
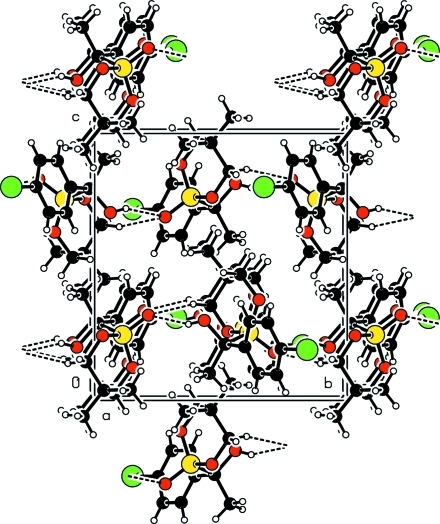
Part of the crystal packing, showing the intermolecular hydrogen bonds as dashed lines.

## supplementary crystallographic information

### Comment

Acyclic and cyclic α-hydroxyphosphonates can be used as very viable intermediates and they are also an attractive class of biologically active compounds (Peng *et al.*, 2007; Liu *et al.*, 2006). In our research work aimed at searching for novel agrochemicals, we attempted to synthesize hydroxyphosphonates according to published literature procedures. Here we report the synthesis and crystal structure of the chiral title compound (I) (Fig. 1). The bond lengths and angles show normal values (Allen *et al.*, 1987). In the crystal structure, the cyclic phosphonate ring adopts a chair conformation.

### Experimental

Hydroxyphosphonate (I) was prepared according to a literature procedure (Zhou *et al.*, 2008). Diethylaluminium chloride (1 mmol) was added to a solution of (*S*,*E*)-2-(adamantan-1-yl)-4-(*tert*-butyl)-6(((1-hydroxy-3-methylbutan-2-yl)imino)methyl)-phenol (1 mmol) in dichloromethane (10 ml). The mixture was stirred at room temperature for 1 h. The ketone (11 mmol) and the cyclic phosphate (10 mmol) were added and the mixture was stirred for 2 h. The reaction was quenched by diluted hydrochloric acid (15:1, *v*/*v*). The pure hydroxyphosphonate was afforded by column chromatography on silica gel (acetone/petroleum ether = 1:2), 71% yield, [α]_D_ = -64.8 ° (c = 0.56, chloroform). Then recrystallization from acetic ester over a period of one week gave colourless crystals of (I).

### Refinement

C-bound H atoms were geometrically positioned (C—H = 0.93–0.98 Å) and refined as riding, with *U*_iso_(H) = 1.2*U*_eq_–1.5*U*_eq_(C). The O-bound H atom was located from a difference Fourier map and refined as riding, with O—H = 0.81 (2) Å and *U*_iso_(H) = 1.5*U*_eq_(O).

### Crystal data

**Table:**

C_13_H_18_BrO_4_P	*F*(000) = 712
*M**_r_* = 349.15	*D*_x_ = 1.549 Mg m^−^^3^
Orthorhombic, *P*2_1_2_1_2_1_	Mo *K*α radiation, λ = 0.71073 Å
Hall symbol: P 2ac 2ab	Cell parameters from 3882 reflections
*a* = 11.0662 (17) Å	θ = 2.5–26.7°
*b* = 11.3149 (18) Å	µ = 2.86 mm^−^^1^
*c* = 11.9609 (19) Å	*T* = 298 K
*V* = 1497.7 (4) Å^3^	Block, colourless
*Z* = 4	0.20 × 0.12 × 0.10 mm

### Data collection

**Table:**

Bruker SMART APEX CCD area-detector diffractometer	2691 reflections with *I* > 2σ(*I*)
Radiation source: fine-focus sealed tube	*R*_int_ = 0.114
graphite	θ_max_ = 28.3°, θ_min_ = 2.5°
φ and ω scans	*h* = −14→14
10072 measured reflections	*k* = −14→14
3627 independent reflections	*l* = −15→12

### Refinement

**Table:**

Refinement on *F*^2^	Secondary atom site location: difference Fourier map
Least-squares matrix: full	Hydrogen site location: inferred from neighbouring sites
*R*[*F*^2^ > 2σ(*F*^2^)] = 0.045	H-atom parameters constrained
*wR*(*F*^2^) = 0.105	*w* = 1/[σ^2^(*F*_o_^2^) + (0.0423*P*)^2^] where *P* = (*F*_o_^2^ + 2*F*_c_^2^)/3
*S* = 0.93	(Δ/σ)_max_ = 0.001
3627 reflections	Δρ_max_ = 0.46 e Å^−^^3^
176 parameters	Δρ_min_ = −0.45 e Å^−^^3^
0 restraints	Absolute structure: Flack (1983), with 1517 Friedel pairs
Primary atom site location: structure-invariant direct methods	Flack parameter: −0.011 (10)

### Special details

**Table:**

Geometry. All e.s.d.'s (except the e.s.d. in the dihedral angle between two l.s. planes) are estimated using the full covariance matrix. The cell e.s.d.'s are taken into account individually in the estimation of e.s.d.'s in distances, angles and torsion angles; correlations between e.s.d.'s in cell parameters are only used when they are defined by crystal symmetry. An approximate (isotropic) treatment of cell e.s.d.'s is used for estimating e.s.d.'s involving l.s. planes.
Refinement. Refinement of *F*^2^ against ALL reflections. The weighted *R*-factor *wR* and goodness of fit *S* are based on *F*^2^, conventional *R*-factors *R* are based on *F*, with *F* set to zero for negative *F*^2^. The threshold expression of *F*^2^ > σ(*F*^2^) is used only for calculating *R*-factors(gt) *etc*. and is not relevant to the choice of reflections for refinement. *R*-factors based on *F*^2^ are statistically about twice as large as those based on *F*, and *R*-factors based on ALL data will be even larger.

### Fractional atomic coordinates and isotropic or equivalent isotropic displacement parameters (Å^2^)

**Table:**

	*x*	*y*	*z*	*U*_iso_*/*U*_eq_	
Br1	−0.07457 (4)	0.16872 (4)	0.69930 (5)	0.0869 (2)	
C1	0.0650 (3)	0.2689 (3)	0.7001 (3)	0.0535 (8)	
C2	0.0982 (3)	0.3216 (3)	0.7973 (3)	0.0533 (8)	
H2	0.0520	0.3113	0.8615	0.064*	
C3	0.2007 (3)	0.3905 (3)	0.8000 (3)	0.0429 (7)	
H3	0.2244	0.4250	0.8671	0.052*	
C4	0.2690 (2)	0.4094 (2)	0.7050 (3)	0.0373 (6)	
C5	0.2317 (3)	0.3561 (3)	0.6058 (3)	0.0498 (8)	
H5	0.2762	0.3679	0.5408	0.060*	
C6	0.1288 (3)	0.2857 (3)	0.6026 (3)	0.0542 (9)	
H6	0.1037	0.2507	0.5362	0.065*	
C7	0.3824 (3)	0.4854 (2)	0.7128 (3)	0.0364 (6)	
C8	0.4129 (4)	0.5472 (3)	0.6033 (3)	0.0557 (9)	
H8A	0.3464	0.5965	0.5813	0.084*	
H8B	0.4277	0.4892	0.5464	0.084*	
H8C	0.4838	0.5949	0.6133	0.084*	
C9	0.5250 (3)	0.4228 (4)	0.9724 (3)	0.0608 (10)	
H9A	0.5186	0.3769	1.0406	0.073*	
H9B	0.4712	0.4902	0.9791	0.073*	
C10	0.6530 (3)	0.4663 (4)	0.9596 (3)	0.0598 (10)	
C11	0.6616 (3)	0.5380 (3)	0.8524 (3)	0.0544 (9)	
H11A	0.6113	0.6079	0.8592	0.065*	
H11B	0.7444	0.5638	0.8419	0.065*	
C12	0.6801 (5)	0.5486 (6)	1.0566 (4)	0.1081 (19)	
H12A	0.6297	0.6174	1.0514	0.162*	
H12B	0.7635	0.5720	1.0540	0.162*	
H12C	0.6644	0.5085	1.1258	0.162*	
C13	0.7422 (4)	0.3633 (4)	0.9547 (5)	0.0885 (15)	
H13A	0.7281	0.3112	1.0167	0.133*	
H13B	0.8233	0.3932	0.9585	0.133*	
H13C	0.7314	0.3208	0.8860	0.133*	
O1	0.36447 (19)	0.56809 (16)	0.80211 (19)	0.0426 (5)	
H1	0.3995	0.6301	0.7874	0.064*	
O2	0.5245 (2)	0.28766 (19)	0.6800 (2)	0.0596 (6)	
O3	0.48668 (19)	0.34977 (18)	0.8773 (2)	0.0497 (5)	
O4	0.6236 (2)	0.4709 (2)	0.7554 (2)	0.0520 (6)	
P1	0.50921 (6)	0.38934 (6)	0.75398 (7)	0.03831 (18)	

### Atomic displacement parameters (Å^2^)

**Table:**

	*U*^11^	*U*^22^	*U*^33^	*U*^12^	*U*^13^	*U*^23^
Br1	0.0855 (3)	0.0813 (3)	0.0938 (3)	−0.0491 (2)	−0.0168 (3)	0.0076 (2)
C1	0.0510 (16)	0.0373 (14)	0.072 (2)	−0.0124 (14)	−0.013 (2)	0.0034 (18)
C2	0.0555 (18)	0.0470 (16)	0.057 (2)	−0.0092 (15)	0.0010 (18)	0.0093 (18)
C3	0.0469 (15)	0.0378 (14)	0.0441 (16)	−0.0061 (13)	−0.0027 (15)	−0.0018 (16)
C4	0.0366 (13)	0.0287 (12)	0.0464 (16)	0.0067 (11)	−0.0042 (14)	−0.0006 (13)
C5	0.0528 (17)	0.0502 (19)	0.0464 (18)	0.0022 (17)	−0.0007 (15)	−0.0032 (16)
C6	0.0547 (18)	0.0492 (18)	0.059 (2)	−0.0037 (18)	−0.0127 (18)	−0.0109 (17)
C7	0.0373 (13)	0.0317 (12)	0.0400 (15)	0.0021 (11)	−0.0015 (13)	−0.0030 (12)
C8	0.064 (2)	0.0477 (17)	0.055 (2)	−0.0051 (19)	0.0061 (18)	0.0061 (16)
C9	0.0599 (19)	0.077 (2)	0.0454 (19)	0.006 (2)	−0.0014 (17)	0.0083 (18)
C10	0.0461 (17)	0.067 (2)	0.067 (2)	0.0070 (18)	−0.0158 (18)	−0.002 (2)
C11	0.0437 (16)	0.0476 (19)	0.072 (2)	−0.0055 (15)	−0.0129 (17)	−0.0052 (18)
C12	0.104 (4)	0.146 (5)	0.074 (3)	−0.010 (4)	−0.031 (3)	−0.029 (4)
C13	0.063 (2)	0.079 (3)	0.124 (4)	0.015 (2)	−0.022 (3)	0.020 (3)
O1	0.0476 (11)	0.0263 (9)	0.0540 (13)	−0.0040 (8)	0.0070 (11)	−0.0088 (10)
O2	0.0618 (13)	0.0404 (11)	0.0768 (17)	0.0148 (12)	−0.0035 (13)	−0.0178 (11)
O3	0.0441 (10)	0.0430 (11)	0.0619 (13)	−0.0006 (11)	−0.0005 (11)	0.0105 (10)
O4	0.0416 (10)	0.0596 (13)	0.0548 (13)	−0.0138 (11)	0.0053 (11)	−0.0026 (12)
P1	0.0339 (3)	0.0299 (3)	0.0511 (4)	0.0027 (3)	0.0014 (3)	−0.0047 (3)

### Geometric parameters (Å, °)

**Table:**

Br1—C1	1.915 (3)	C9—C10	1.508 (5)
C1—C2	1.357 (5)	C9—H9A	0.9700
C1—C6	1.377 (5)	C9—H9B	0.9700
C2—C3	1.377 (4)	C10—C12	1.518 (7)
C2—H2	0.9300	C10—C11	1.520 (5)
C3—C4	1.382 (5)	C10—C13	1.528 (5)
C3—H3	0.9300	C11—O4	1.449 (4)
C4—C5	1.393 (5)	C11—H11A	0.9700
C4—C7	1.524 (4)	C11—H11B	0.9700
C5—C6	1.391 (5)	C12—H12A	0.9600
C5—H5	0.9300	C12—H12B	0.9600
C6—H6	0.9300	C12—H12C	0.9600
C7—O1	1.434 (4)	C13—H13A	0.9600
C7—C8	1.522 (5)	C13—H13B	0.9600
C7—P1	1.842 (3)	C13—H13C	0.9600
C8—H8A	0.9600	O1—H1	0.8200
C8—H8B	0.9600	O2—P1	1.461 (2)
C8—H8C	0.9600	O3—P1	1.562 (2)
C9—O3	1.469 (4)	O4—P1	1.566 (2)
			
C2—C1—C6	121.7 (3)	H9A—C9—H9B	107.9
C2—C1—Br1	118.9 (3)	C9—C10—C12	107.9 (4)
C6—C1—Br1	119.4 (3)	C9—C10—C11	108.6 (3)
C1—C2—C3	119.5 (3)	C12—C10—C11	107.8 (4)
C1—C2—H2	120.3	C9—C10—C13	111.3 (3)
C3—C2—H2	120.3	C12—C10—C13	111.7 (4)
C2—C3—C4	121.3 (3)	C11—C10—C13	109.6 (3)
C2—C3—H3	119.4	O4—C11—C10	112.2 (3)
C4—C3—H3	119.4	O4—C11—H11A	109.2
C3—C4—C5	118.1 (3)	C10—C11—H11A	109.2
C3—C4—C7	119.2 (3)	O4—C11—H11B	109.2
C5—C4—C7	122.7 (3)	C10—C11—H11B	109.2
C6—C5—C4	120.9 (3)	H11A—C11—H11B	107.9
C6—C5—H5	119.5	C10—C12—H12A	109.5
C4—C5—H5	119.5	C10—C12—H12B	109.5
C1—C6—C5	118.4 (3)	H12A—C12—H12B	109.5
C1—C6—H6	120.8	C10—C12—H12C	109.5
C5—C6—H6	120.8	H12A—C12—H12C	109.5
O1—C7—C8	111.8 (2)	H12B—C12—H12C	109.5
O1—C7—C4	107.5 (2)	C10—C13—H13A	109.5
C8—C7—C4	112.9 (3)	C10—C13—H13B	109.5
O1—C7—P1	106.92 (19)	H13A—C13—H13B	109.5
C8—C7—P1	109.4 (2)	C10—C13—H13C	109.5
C4—C7—P1	108.11 (18)	H13A—C13—H13C	109.5
C7—C8—H8A	109.5	H13B—C13—H13C	109.5
C7—C8—H8B	109.5	C7—O1—H1	109.5
H8A—C8—H8B	109.5	C9—O3—P1	121.6 (2)
C7—C8—H8C	109.5	C11—O4—P1	123.5 (2)
H8A—C8—H8C	109.5	O2—P1—O3	111.41 (14)
H8B—C8—H8C	109.5	O2—P1—O4	112.12 (14)
O3—C9—C10	112.1 (3)	O3—P1—O4	106.72 (12)
O3—C9—H9A	109.2	O2—P1—C7	112.97 (13)
C10—C9—H9A	109.2	O3—P1—C7	107.47 (13)
O3—C9—H9B	109.2	O4—P1—C7	105.74 (13)
C10—C9—H9B	109.2		
			
C6—C1—C2—C3	−2.4 (5)	C12—C10—C11—O4	−173.3 (4)
Br1—C1—C2—C3	177.7 (2)	C13—C10—C11—O4	65.0 (4)
C1—C2—C3—C4	1.6 (5)	C10—C9—O3—P1	−47.2 (4)
C2—C3—C4—C5	−0.3 (4)	C10—C11—O4—P1	43.1 (4)
C2—C3—C4—C7	−179.4 (3)	C9—O3—P1—O2	150.2 (2)
C3—C4—C5—C6	−0.2 (4)	C9—O3—P1—O4	27.5 (3)
C7—C4—C5—C6	178.8 (3)	C9—O3—P1—C7	−85.6 (2)
C2—C1—C6—C5	1.8 (5)	C11—O4—P1—O2	−148.2 (2)
Br1—C1—C6—C5	−178.3 (2)	C11—O4—P1—O3	−25.9 (3)
C4—C5—C6—C1	−0.5 (5)	C11—O4—P1—C7	88.3 (3)
C3—C4—C7—O1	−28.2 (3)	O1—C7—P1—O2	169.99 (19)
C5—C4—C7—O1	152.7 (3)	C8—C7—P1—O2	−68.8 (2)
C3—C4—C7—C8	−152.0 (3)	C4—C7—P1—O2	54.6 (2)
C5—C4—C7—C8	29.0 (4)	O1—C7—P1—O3	46.7 (2)
C3—C4—C7—P1	86.8 (3)	C8—C7—P1—O3	167.9 (2)
C5—C4—C7—P1	−92.2 (3)	C4—C7—P1—O3	−68.8 (2)
O3—C9—C10—C12	175.4 (4)	O1—C7—P1—O4	−67.0 (2)
O3—C9—C10—C11	58.9 (4)	C8—C7—P1—O4	54.2 (2)
O3—C9—C10—C13	−61.8 (4)	C4—C7—P1—O4	177.5 (2)
C9—C10—C11—O4	−56.7 (4)		

### Hydrogen-bond geometry (Å, °)

**Table:**

*D*—H···*A*	*D*—H	H···*A*	*D*···*A*	*D*—H···*A*
O1—H1···O2^i^	0.82	2.01	2.780 (3)	156.
C9—H9B···O1	0.97	2.58	3.163 (4)	119.
C11—H11A···O2^i^	0.97	2.57	3.517 (4)	165.

Symmetry codes: (i) −*x*+1, *y*+1/2, −*z*+3/2.
